# Fungal Infections as an Uprising Threat to Human Health: Chemosensitization of Fungal Pathogens With AFP From *Aspergillus giganteus*

**DOI:** 10.3389/fcimb.2022.887971

**Published:** 2022-05-25

**Authors:** Kavitha Dhandapani, Karthiga Sivarajan, Ramya Ravindhiran, Jothi Nayaki Sekar

**Affiliations:** Department of Biochemistry, Biotechnology and Bioinformatics, Avinashilingam Institute for Home Science and Higher Education for Women, Tamil Nadu, India

**Keywords:** chemosensitization, antifungal drugs, natural compounds, augmentation, fungicidal activity, *Aspergillus giganteus*, AFP

## Abstract

Occurrence and intensity of systemic invasive fungal infections have significantly risen in recent decades with large amount of mortality and morbidity rates at global level. Treatment therapy lies on the current antifungal interventions and are often limited due to the emergence of resistance to antifungal agents. Chemosensitization of fungal strains to the conventional antimycotic drugs are of growing concern. Current antifungal drugs often have been reported with poor activity and side effects to the host and have a few number of targets to manifest their efficacy on the pathogens. Indiscriminately, the aforementioned issues have been easily resolved by the development of new intervention strategies. One such approach is to employ combinational therapy that has exhibited a great level of inhibitions than that of a single compound. Chemosensitization of pathogenic mycoses to commercial antifungal drugs could be drastically enhanced by co-application of chemosensitizers along with the conventional drugs. Chemosensitizers could address the resistance mechanisms evolved in the pathogenic fungi and targeting the system to make the organism susceptible to commercially and clinically proven antifungal drugs. However, this strategy has not been overreached to the greater level, but it needs much attention to fight against not only with the pathogen but combat the resistance mechanisms of pathogens to drugs. Natural compounds including plant compounds and microbial proteins act as potential chemosensitizers to break the resistance in mycoses. *Aspergillus giganteus*, a filamentous fungus, is known to produce a cysteine rich extracellular protein called as antifungal protein (AFP). AFP has shown enhanced efficacy against several filamentous and non-filamentous fungal pathogens. On the basis of the reported studies on its targeted potential against pathogenic mycoses, AFP would be fabricated as a good chemosensitizer to augment the fungicidal efficacy of commercial antimycotic drugs. This paper reviews on breakthrough in the discovery of antifungal drugs along with the resistance patterns of mycoses to commercial drugs followed by the current intervention strategies applied to augment the fungicidal potential of drugs.

## Background

Fungal infections, predominantly aspergillosis, candidiasis, and cryptococcosis, are of main global concern because of their causative agents and resistance toward commercial antifungal drugs. Development of antimycotic drugs have lagged behind by the fact that fungi are eukaryotes, known to have a similar and closer evolutionary relationship to humans. Unlike other pathogens, they own a similar attributes including biochemical, genetic, and cellular biology common to humans (Campbell et al., 2012). Major antifungal drugs fall under three categories: azoles, polyenes, and echinocandins, whereas azoles and polyenes were clinically approved and applied for the treatment before 1980s. In 20th century, third-category drugs, namely, echinocandins (Caspofungin) was introduced and used in routine clinical purposes to treat fungal infections (Roemer and Krysan, 2014). Although these drugs are effectively applied in clinical area on systemic and invasive fungal infections, the emergence of antifungal resistance make fungal diseases a global human threat, especially in immunocompromised individuals (McCarthy et al., 2017). The increased incidence of fungal diseases may require an adequate level of antimycotic chemotherapy, ultimately leading to the overreaching problem of antifungal resistance. The development of resistance involves a vicious cycle of events that results in emergence of more resistant strains and thus, in turn, promotes the reinforcement of therapeutically intractable fungal diseases.

*Aspergillus* species are frequent causative agents of invasive fungal infections, with aspergillosis mainly affecting persons with hematological malignancies and after solid-organ or hematopoietic stem cell transplantations. Frontline antimycotic drugs therapy for the treatment of aspergillosis are voriconazole (VOR), liposomal amphotericin B lipid complex (AmB), posaconazole (POS), micafungin (MICA), itraconazole (ITR), anidulafungin (ANI), and caspofungin (CAS) (Kim et al., 2010). Since the expansion of resistance to the conventional antimycotic drugs is of global concern, there is a pressing effort to make the pathogens susceptible for antifungal drugs. Indeed, further issue is the stagnation in the development of effective and novel antifungal drugs for the treatment (Kim et al., 2016). Chemosensitization by natural compounds is a new strategy to deal with the aforementioned issue will enhance the efficacy of conventional antimycotics. Over three decades, natural compounds with no toxicity are currently tested to augment the antifungal efficacy. Chemosensitizing agents cannot be applied as alone for the effective treatment, when co-applied with the commercial antibiotics, might countermands the antifungal resistance and augment the efficacy to treat the infamy fungal infections (Kim et al., 2019).

Here, we comment on the recent chemosensitizing efforts as alternative strategy for the control and treatment of fungal infections by conventional antimycotic drugs. The review primarily encapsulated with the antifungal-resistant mechanism evolved by fungal pathogens followed by the role of chemosensitizers in the control of fungal infections.

## Breakthrough in the Discovery of Antimycotic Drugs

**Figure 1:**
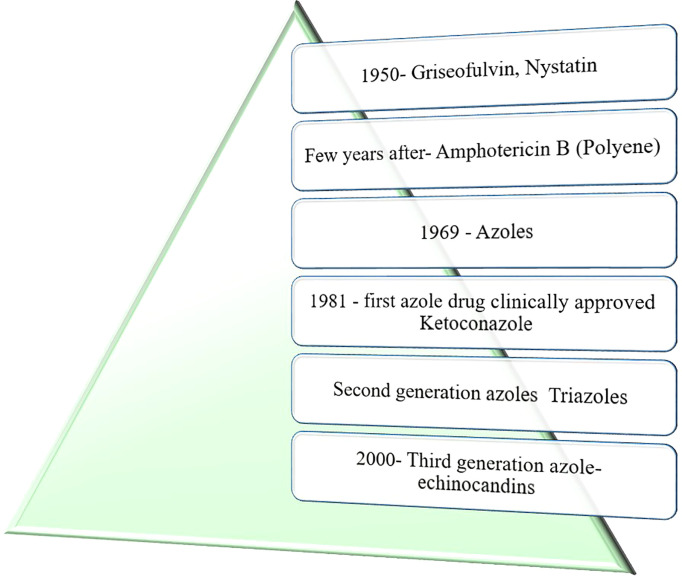
Development of antimycotic drugs.

In the early 1950s, no fungal-specific antimicrobials were developed but were possible after two decades of penicillin discovery. **Figure 1** shows the evolution of antimycotic drugs for the control of fungal infections. Griseofulvin (polyketide) from *Penicillium griseofulvum* and nystatin (polyene) from *Streptomyces* bacterium were the initial antimycotic drugs isolated. Grieseofulvin follows the mechanism of disrupting the mitotic spindle microtubules, thus impeding the cellular division in fungal pathogens. Nystatin has the ability to form a hydrogen bonds with ergosterol and creating a pore on the plasma membrane for the leakage of cellular materials in fungi. Ergosterol is the fungal specific sterol responsible for the maintenance of structural integrity of fungi. Nystatin is advantageous over griseofulvin in clinical treatment. Griseofulvin has nephrotoxicity limiting to internal applications and used for dermatophytic and oropharyngeal infections (Panda et al., 2005; Campbell et al., 2012).

## Importance of Polyenes in Intractable Fungal Infections

Wide spectrum of antimycotic drugs have emerged since, with nystatin able to disrupt the cell membrane integrity of the pathogen. After that, two kinds of polyene from *Streptomyces nodosus* were isolated, namely amphotericin A (AmA) and Amphotericin B (AmB), based on their amphoteric properties. A tetraene amphotericin A has the antifungal spectrum similar to nystatin. A heptaene AmB was found to have a greater extent of antimycotic activity than nystatin, which is considered as the milestone in the antifungal therapy. More than one and a half decades of research has been expended to unravel the chemical nature and structure of AmB. In total, six number of polyene antifungals have been exploited in antifungal treatment, namely, amphotericin B, nystatin, natamycin, candicidin, methyl partricin, and trichomycin. Among which, only three polyenes have been used widely in current antifungal therapies such as AmB for systemic invasive fungal infections, nystatin for mucosal (oral infections or vulvovaginal candidiasis), and natamycin for ophthalmic infections. Since AmB is not soluble in water due to its amphipathic properties, researchers have developed various forms of AMB (lipid, liposomal form, and complex) to minimize their side effects and make it effective in treating fungal infections with less toxic and side effects to the human (Roemer and Krysan, 2014).

There are four models of mode of polyene actions: pore forming model, surface adsorption model, sterol sponge model, and oxidative damage model. In all the proposed models, targeting the ergosterol is the key to exert their antifungal activity. Ergosterol is the important cellular components in the fungal cell actively participating in several cellular functions including regulation of membrane proteins, cell division, endocytosis, cell signaling, and membrane fluidity. AmB binds to ergosterol, apart from binding to other sterols including cholesterol, lanosterol, and others. There are three kinds of non-covalent interactions that have been involved in the interaction between AmB and ergosterol: hydrogen bonds, van der Waals interactions, and π-π electronic interactions. In the pore forming model, the drug and ergosterol interaction forms an ion-like complex that releases ions and small molecules out of the cells to cell death. In the AmB oriented on the plasma membrane based on their amphipathic nature, the hydrophobic tails are interacting with the ergosterol and eventually enter into the lipid environment, whereas the hydrophilic heads might form an aqueous channel. On the basis of AmB function, it is possible to make two types of pores on the membrane: complete pore on the membrane consists of two polyenes and a half-pore containing only one polyene ring. Both the pores have the ability to change the conformational changes on the plasma membrane (Carolus et al., 2020).

The next models, namely, surface adsorption model and sterol sponge model, destabilize the plasma membrane, leading to disturbances in the cellular process such as endocytosis and regulation of membrane protein. Several studies have claimed that AmB follows an additional model of action toward oxidative damage. In *Cryptococcus neoformans*, addition of AmB causes cell becomes metabolically inactive and produces a strong oxidative damage to contribute cell death *via* lipid peroxidation processes. However, the exact mechanism of oxidative stress in the fungal cells is not clear. The process is assumed that binding of polyenes to the plasma membrane triggers oxidative stress, eventually leading to the apoptotic events in the cells including ROS production. AmB is known to auto-oxidize and generates free radicals; thus, the antifungals itself produces strong oxidative stress inside the cell (Carolus et al., 2020).

## Essential Development of Azoles as Potential Antifungal Drugs

Azoles, the next category of approved antimycotic drugs, have been developed in the year 1969. It interferes in the ergosterol biosynthesis and inhibits the enzyme 14α-sterol demethylase. Various azoles have been bifurcated into imidazoles and triazoles. Major imidazoles are miconazole, bifonazole, clotrimazole, and ketoconazole but are largely limited to systemic use because of their hepatotoxicity. In 1981, ketoconazole was the first clinically approved azole drug for the systemic use, but it has less effective against various fungal pathogens (Campbell et al., 2012).

The second group of azoles, triazoles, have much affinity toward the enzyme 14α-sterol demethylase and interfere with ergosterol synthesis, which, in turn, inhibit the fungal growth effectively than imidazoles. The first approved triazole is itraconazole followed by fluconazole. The three triazoles—voriconazole, posaconazole, and ravuconazole—have been accepted and used for systemic infections. During early 2000, the third-generation drug, echinocandins, had evolved. Caspofungin is the first approved antifungal echinocandin drug for the treatment of fungal infections and was well tolerated by the patients. Micafungin and anidulafungin have also been accepted, and echinocandins have been reported to have minimal side effects than polyenes and azoles (Lass-Flörl, 2011).

## Early 2000 Revolution in Antimycotic Drug Discovery

**Figure 2:**
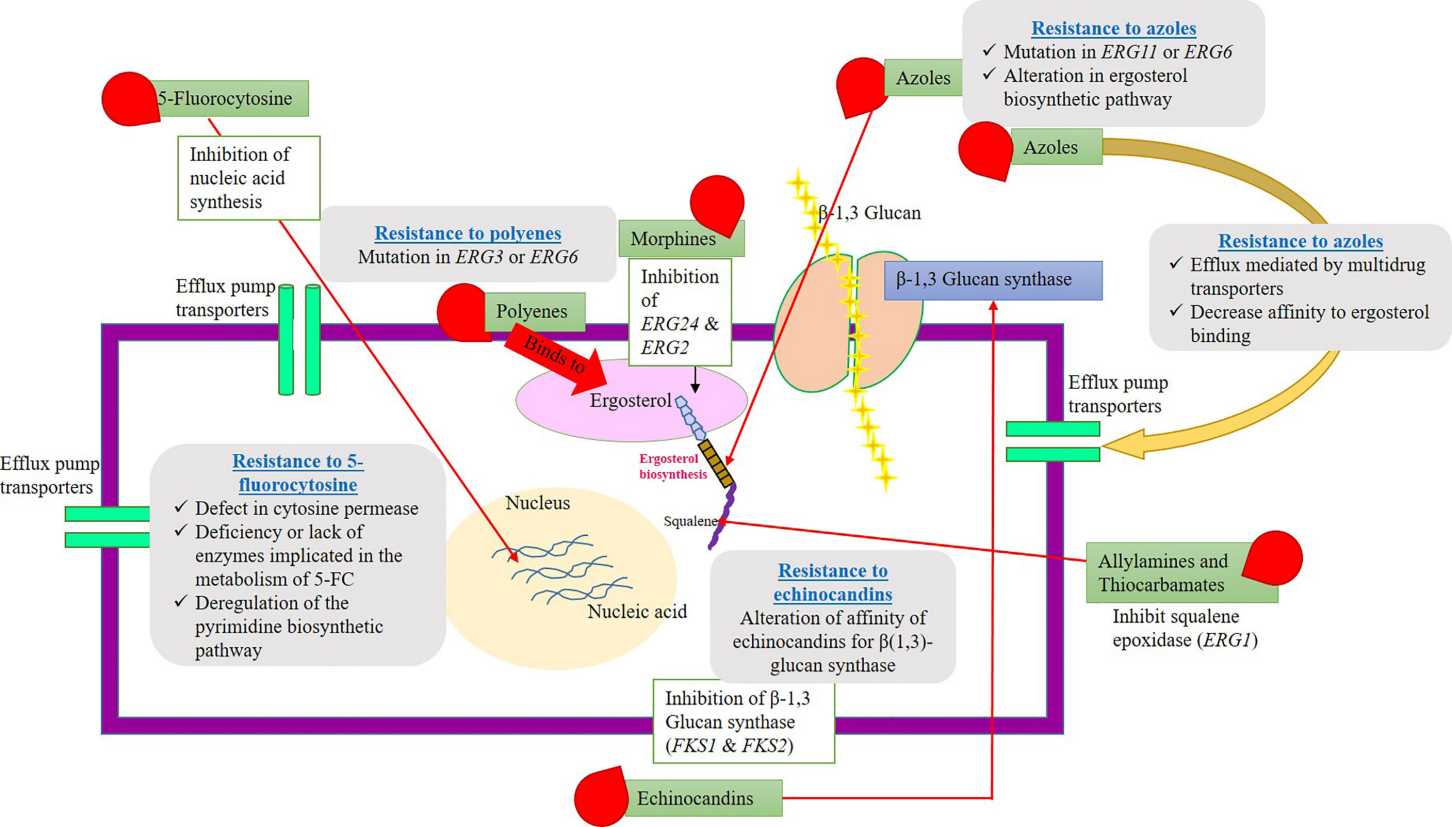
Schematic representation of fungal cell wall, antimycortic drugs, possible antifungal activity (target site), and resistance to commercial antifungal drugs. The picture explains various antifungal compounds and their target site on the pathogenic fungi. Along with that, the plausible resistance mechanisms evolved by the fungal species to clinically approved antimycotic drugs have clearly depicted. Resistance gene/proteins/channels might be antifungal targets for the development of novel antimycotic compounds or alternative chemosensitization/drug repurposing approaches.

5-fluorocytosine (5-FC) resistance can be very intrinsic and nearly 7% to 8% *Candida* species and filamentous fungi (*Aspergillus* and dermatophytes) have developed a resistance toward 5-FC. Nearly, a large number of fungi are known to be susceptible to polyenes drugs. Few of them, namely, *Candida glabrata*, *Scedosporium prolificans*, and *Aspergillus terreus*, have evolved resistance to polyenes (Revie et al., 2018). In the early 1990s, the azole drug resistance has been an alarming threat, and increased incidence of fungal infections was noted. However, the development in the field of antimycotic drug therapy has stabilized the issues. Echinocandins have been introduced in the year 2000, and the resistance to these drugs was rare events. Several *Candida* and *Aspergillus* species are known to be susceptible to echinocandins, but *Cryptococcus neoformans* shows strong resistant to these drugs. The mechanism of echinocandins is to disrupt the cell wall synthesis by inhibiting the enzyme β(1–3)-glucan synthase (Sanglard and White, 2006). The resistance mechanism has been developed due to the alteration in the composition of cell wall components in *Cryptococcus neoformans*. The antifungal drugs, their mode of action, and their targets along with their resistance mechanisms are explained in **Figure 2**.

## Resistance Mechanism to Common Antimycotic Drugs

**Figure 3:**
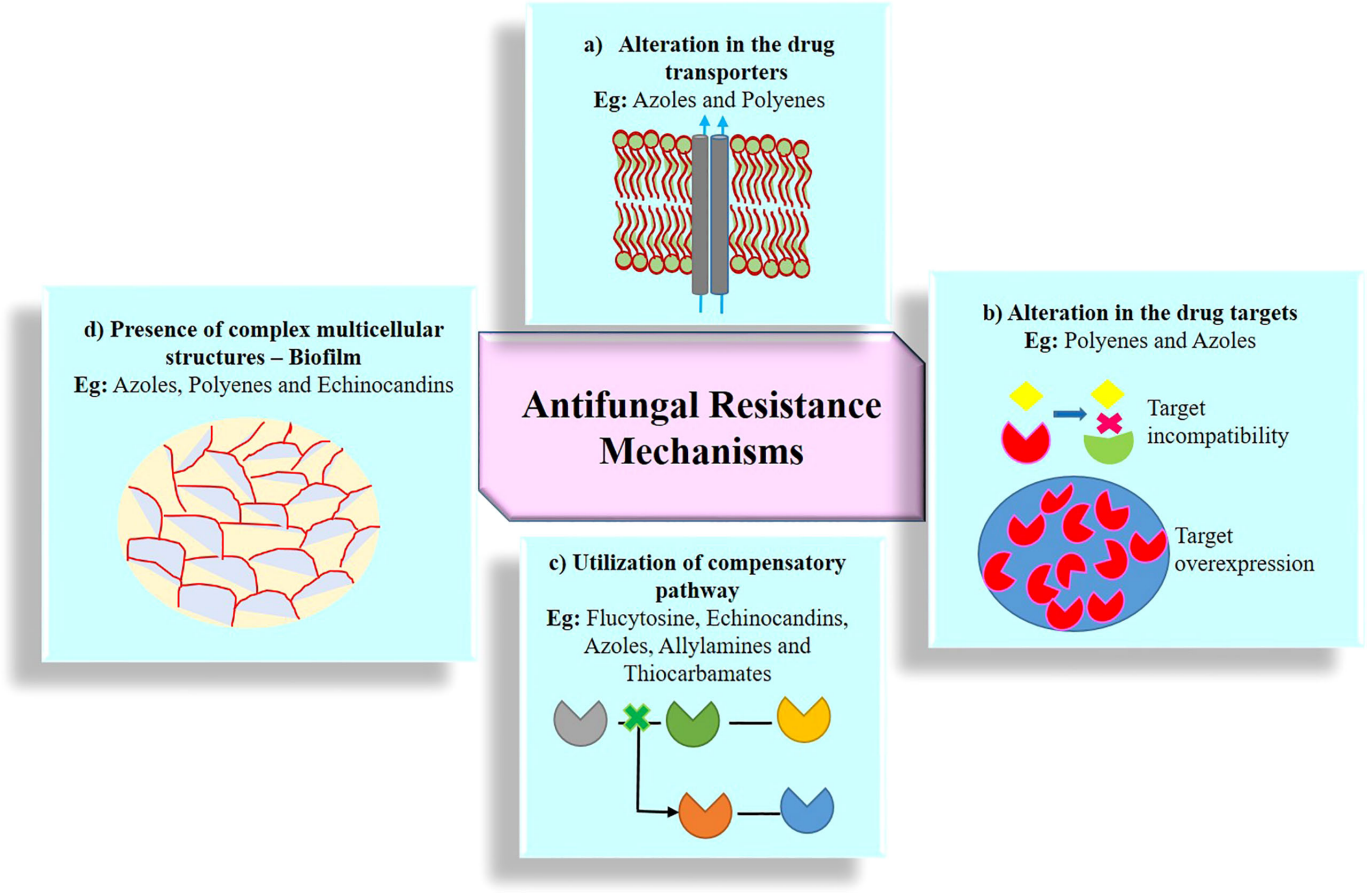
Emergence of antifungal resistance through various mechanisms. **(A)** Alteration in the drug transporters reduce the accumulation of drug molecules in the cell and expel the drugs out of the cell *via* efflux protein transporter; two kind of major efflux pump transporters are ABC (ATP-binding cassette) protein transporters and MFS (major facilitator superfamily) protein transporters; **(B)** alteration in the drug targets substantially reduces the affinity of the drug for its targets by structural modification, mutation, upregulation, downregulation, and overexpression of drug targets; **(C)** utilization of compensatory pathways, ergosterol biosynthesis pathway alteration, and plasma membrane composition variation; **(D)** presence of complex multicellular structures, formation of biofilm, and polymicrobial biofilms (bacterial species often associated with one or more fungal strains).

Resistance to antimycotic drugs is a significant threat to human. Resistant mechanisms evolved by fungal pathogens fall under different categories including (i) alterations of transporters, (ii) alteration of targets, (iii) utilization of compensatory pathways, and (iv) presence of complex multicellular structures. **Figure 3** shows the antimicrobial resistance in fungal pathogens.

## Alterations of Transporters

Two different efflux pumps exists in fungal pathogens, namely, ABC (ATP-binding cassette) protein transporters and MFS (major facilitator superfamily) protein transporters. Nucleotide-binding domains (NBD) and transmembrane domains (TMD) are the two main domains found in ABC proteins. NBD involves in binding and hydrolysis of ATP providing energy for the translocation of substrates, whereas TMD contributes to a substrate channel *via* membrane. Two NBD and two TMD domains have been reported in several fungal ABC transporters. MFS protein transporters are generally referred to as proton antiporters. They can utilize the electrochemical potential and proton motive force across the cell membrane for the translocation of substrates. They do not contain NBD but has TMD.

MFS transporter is the second class of transporters involved in the development of resistance in several fungi. It is found in almost all kingdom and involved in symport, antiport, and uniport of several substrates in and out of the cell. It exerts antifungal resistance by proton antiport and has classified into two major groups: a) the transporter: H+ antiporter-1 family, it has 12 transmembrane α-helix (TMS); b) the transporter: H+ antiporter-2 family, it has 14 transmembrane α-helix (Prasad and Rawal, 2014; Esquivel et al., 2020).

*Aspergillus fumigatus* is responsible for severe invasive aspergillosis in host especially, in immunocompromised individuals. Azole drugs (ITR, VOR, and POS) and echinocandins are the frontline antifungals employed in the treatment of invasive aspergillosis. Azoles directly interfere in the ergosterol pathway by inhibiting the enzyme 14-α demethylase, a key enzyme in ergosterol biosynthesis (Pfaller, 2012; Cowen et al., 2015). Important resistant mechanism in various pathogens is the ability of several membrane protein families to mediate efflux of drug compounds. This kind of resistant involves ABC transporters and MFS transporters that are known to upregulate in the azole-resistant fungal pathogens. Novel genes in *Aspergillus fumigatus* involved in azole resistance have been identified by developing a functional complementation system, and their cDNAs are expressed in *S. cerevisiae* model. Among the several genes including *atrF* and *atrl* of ABC transporters and *mdrA* of MFS transporters, *atrl* gene is responsible for the resistance toward itraconazole and voriconazole. However, *mdrA* shows resistance only to voriconazole in *S. cerevisiae* model system. Further, targeting the genes responsible for the azole resistance by chemosensitizing approach might make the pathogens susceptible to azole drugs (Meneau et al., 2016). ASP2397, a natural compound tested on *in vivo Aspergillus fumigatus* mouse infection model, exerted maximum fungicidal activity compared to that of commercial antimycotic drugs, namely, amphotericin B, voriconazole, posaconazole, itraconazole, and fluconazole (Nakamura et al., 2019).

Randomly, 50% of the fungal incidence were reported with *Candida* spp. among which *Candida parapsilosis* is of major global concern. Azoles are the common antimycotic drugs prescribed for the control and treatment of candidiasis but the organisms have developed resistance to azoles to sustain in the host (Silva et al., 2011; Ding et al., 2018). Berkow et al. have studied about the genes involved in the azole-resistant mechanisms including drug transporters (*CDR1* and *MDR1*) and ergosterol biosynthesis (*ERG3* and *ERG11*) (Berkow et al., 2015). Conclusively, the studies suggested that genes encoded for *cdr1*, *mdr 1*, and *erg11* are directly involved in the formation of azole resistance in *Candida parapsilosis*. Aforementioned studies have recommended that these putative genes are the suitable targets for the chemosensitization strategies.

Nascimento et al. have analyzed the itraconazole-resistant mechanisms in *Aspergillus fumigatus* involving two major efflux protein (MDR) *AfuMDR3* and *AfuMDR4*. They showed prominent changes in their expression level in itraconazole resistant. Among them, *AfuMDR3* belongs to MFS transporters, whereas *AfuMDR4* was a typical member of the ABC superfamily. Overexpression of either one or both the efflux transporter genes is responsible for the enhanced itraconazole resistance in *Aspergillus fumigatus* isolates, and, thus, in turn, they are the suitable drug targets to chemosensitize or finding novel drugs for the treatment of aspergillosis. Ferreira et al. have also found that overexpression of drug efflux pump transporters is the main reason for the development of drug resistance in *Aspergillus fumigatus*, where five ABC transporters genes and MFS transporters genes contribute to voriconazole-resistant development. *cdr1B*, ABC transporter efflux pump genes, led to the emergence of azole resistance in *Aspergillus fumigatus* (Paul et al., 2013). Overexpression of drug efflux pump protein MFS genes, *AflMDR1* and *AflMDR2*, is responsible for cilofungin resistance in *A. flavus* (Tobin et al., 1997), and overexpression of ABC transporters genes, *atrA*, *atrB*, *atr*C, and *atrD*, leads to azole resistance in *Aspergillus nidulans* (Semighini et al., 2002).

## Alteration of Drug Targets and Utilization of Compensatory Pathways

In addition to the overexpression of efflux pump transporters, the mutation of drug targets including enzymes responsible for ergosterol or β-1,3 glucan synthesis might induce potential resistance in fungal pathogens to common antifungals. Generally, 25 known enzymes are involved in the regulation of ergosterol biosynthesis in fungal cell. It is denoted by *ERG* enzymes where any mutation, alteration, and gene deletion of these enzymes lead to the resistance of fungi to various antimycotic drugs (Bhattacharya et al., 2018). *ERG1*, *ERG2*, *ERG6*, *ERG11* alterations lead to the azole resistance and amphotericin B resistance in *Candida albicans* (Hull et al., 2012). Point mutations at the target site (drug binding site) have reduced the affinity and thus, in turn, showed the inability of the drug to bind with targets. CYP51A encodes the enzyme, namely, lanosterol 14α-demethylase, and mutations at this gene have been recorded for the development of azole resistance in *Aspergillus fumigatus* (Fuentefria et al., 2018). Y136F substitution on this gene has also been noted with the decreased susceptibility to fluconazole and voriconazole (Scorzoni et al., 2017). These point mutations have particularly constraint-specific amino acid substitutions on the target site (enzyme). *Erg11* has altered by point mutation in *Candida albicans*, causing severe resistance toward triazole drugs. Same kind of resistant patterns has been observed in *Aspergillus fumigatus* and *Cryptococcus neoformans*. Mutation on glucan synthase enzyme (*FKS1*) has significantly enhanced the resistance to echinocandins antifungal drugs. A single–amino acid substitution in *FKS1* gene largely contributes the drastic reduction in the susceptibility of *Candida parapsilosis*, *Candida metapsilosis*, and *Candida orthopsilosis* to echinocandins (Garcia-Effron et al., 2008). 5-flucytosine is generally imported by the enzyme, cytosine permease, and encoded by the gene *Fcy2p* followed by the deamination process, utilizing the cytosine deaminase enzyme (*Fcy1p*) and finally converted to 5-fluorouridine (5FU). With the action of phosphoribosyltransferase (*Fur1p*), 5-fluorouridine is converted to 5FU monophosphate. In activation or mutation of any of the abovementioned genes confer a strong resistance to 5-flucytosine (Chapeland-Leclerc et al., 2005; Papon et al., 2007). Mutation in the *ERG3* gene is responsible for the formation of 14*α*-methyl-3,6-diol from 14*α*-methyl-fecosterol and eventually, accumulating the precursors, can replace the ergosterol and utilize the different pathway. Ultimately, it leads to the strong resistance toward commercial antibiotics in *Candida* sp. (Vandeputte et al., 2012).

Higher activity of enzymes such as catalase and superoxide dismutatse (antioxidant system) along with the intense stress response *via* heat-shock proteins (Hsp70 and Hsp90) in fungal cell resulted in the increased drug resistance in *Aspergillus terreus* (Posch et al., 2018). The key enzyme in ergosterol biosysnthesis is 14-α-demethylase (*CYP51B*), and their mutation and other *ERG* alterations contribute to the greater resistance to 5-flucytosine drugs in *Candida* sp. (Costa et al., 2015). Other important features are overexpression of ABC pump transporter genes *cdr1*, *cdr2*, and *mdr1* that efflux azole drugs out of the fungal cell and create a greater resistance mechanism, whereas *AtrF*, *MDR3*, and *MDR4* and their overexpression lead to azole resistance in *Aspergillus fumigatus* (Cowen et al., 2015). Often, the biofilm formation leads to triazole resistance in *Candida albicans*. Alterations in β-glucan synthase gene (amino acid substitutions) *FKS1* and *FKS2* contribute for echinocandins drug resistance in several fungal pathogens (Rodrigues et al., 2018). Thickened cell wall by increased synthesis of 1,3,β-D-glucans shows relatively greater drug resistance in *Candida tropicalis* (Mesa-Arango et al., 2016).

## Presence of Complex Multicellular Structures

In general, biofilms are referred as surface attached microbial communities surrounded by self-produced polymer matrix. Infectious organisms might develop a biofilm on abiotic surfaces including implanted medical devices, catheters, lenses, and pacemakers that contribute greater resistance to most of the antifungals and pose a serious threat to control and treat the fungal infections (Sardi et al., 2014). For example, 80% of all infections were associated with the biofilm-mediated fungal infections in patients. One of the important resistant mechanism in *Candida* sp. is forming the biofilm-mediated infections that strongly confer the antifungal resistance to several commercial antibiotics. Both *Candida* and non-*Candida* species including *Candida glabrata*, *Candida tropicalis*, and *Candida krusei* have the ability to form a clinically relevant biofilm in patients (Benakanakere and Kinane, 2012). In molecular aspects, formation of biofilm associated with the transcriptional regulatory genes, *TEC1*, *BCR1*, and *EFG1*, are the important genes responsible for the strong growth of biofilms in *Candida albicans*. Agglutinin-like sequence (ALS) has eight members from *ALS1* to *ALS8* that code for glycophosphatidylinositol-anchored cell surface glycoproteins. Among them, *ALS3* has been actively associated with the biofilm formation and contributes for drastic reduction in susceptibility to antifungals (Murciano et al., 2012). Moreover, ALS, other enzymes, namely, proteases (SAPs), lipase (LIP), and phospholipases (PLB), are contributing to the biofilm development in *C. albicans* and, thus, increased resistance evolved to various antimycotic drugs (Mayer et al., 2013).

With the aforementioned clinical data, targeting the resistance mechanism of pathogenic fungi has deserved much attention by chemosensitization approaches utilizing natural compounds.

## Sensitization of Fungal Pathogens to Natural Chemosensitizers

Development of resistance of the fungal species against numerous antifungal drugs is of growing concern in the scientific community. The rate of increasing drug resistance is greater than that of novel compounds introduced for the treatment of fungal infections. Considering the dearth of current conventional antimycotic drugs, screening of molecules to enhance the fungicidal activity has been prioritized. Paradoxically, against resistant fungal strains, chemosensitization is an appealing alternative approach to make the same drug susceptible to the fungal pathogens. There are various synonymous words for chemosensitizers including enhancers, synergizers, augmenting agents, potentiators, sensitizers, and even others. We need to focus on the compounds that are also not toxic to the patients. In ancient era, herbal medicine has been widely applied for treating mycoses. Natural compounds used as chemosensitizer are initially referred as “pharmacologic circumvention of multidrug resistance”, where *vinca* alkaloids potentially inhibit the efflux pump overexpression for susceptibility of anticancer drugs (Ford and Hait, 1994). By considering the facts of emergence of antifungal resistance to commercial antifungal drugs, researchers have spurred on an endeavor to look for new strategies and natural compounds in antifungal chemotherapy. Wide spectrum of natural compounds acts as a chemosenitizer to augment fungicidal activity and is tabulated in **Table 1**.

**Table 1:** Natural chemosensitizer along with the commercial antimycotic drugs to augment fungicidal activity against human mycoses.

Chemosensitizer	Antimycotic Drugs	Pathogenic Fungi	Function	Reference
Octyl gallate	Caspofungin	*Aspergillus fumigatus*, *Aspergillus flavus*, *Aspergillus parasiticus*, *Penicillium expansum*, *Penicillium glabrum*, *Penicillium chrysogenum*, *P. griseofulvum*, *P. italicum*	Sensitize cell wall integrity and antioxidant systems of filamentous fungi	Kim et al., 2018
Retigeric acid B	Fluconazole, ketoconazole, itraconazole	*C. albicans*	Targeting the efflux pump transporters	Chang et al., 2012
Anethole	Polygodial, amphotericin B, miconazole	*C. albicans*	Synergistic activity	Kubo and Himejima, 1991; Lee and Kim, 1999
Essential oil of *Ocimum sanctum*	Fluconazole, ketoconazole	*Candida* sp.	Interferes with chitin synthase enzyme	Amber et al., 2010
Schinol	Itraconazole, amphotericin B, trimethoprim-sulfamethoxazole	*Paracoccidioides brasiliensis*	Interferes with the cell wall synthesis or assembly	Johann et al., 2010
Cinnamaldehyde analogs	Fluconazole	*Candida* sp.	Overcome resistance/H+-efflux	Shreaz et al., 2011; Shreaz et al., 2013
Anisaldehyde analogs			Overcome resistance/ATPase proton pump	
Thymol	Ketoconazole, fluconazole, amphotericin B	*Aspergillus fumigatus*	Target the cell wall/cell membrane integrity pathway	Kim et al., 2008
Natural benzaldehyde and its structural analogs	Antimycin A, carboxin	*Aspergillus fumigatus*, *Aspergillus flavus*, *Aspergillus terreus*, *Penicillium expansum*	Disrupt cell wall/cell membrane integrity	Kim et al., 2011
Clomiphene Toremifene Raloxifene Ospemifene, Resveratrol Cis-stilbene	Itraconazole, fluconazole	*Candida albicans*, *Candida auris*, *Cryptococcus neoformans*, *Aspergillus fumigatus*	Inhibiting efflux transporters-ABC and MFS membrane transporters	Eldesouky et al., 2020a
Pitavastatin	Fluconazole	*Candida albicans*, *Candida glabrata Candida auris*	Interfere with the biofilm formation	Eldesouky et al., 2020b
2,3-dihydroxy-benzaldehyde, thymol or salicylaldehyde	Amphotericin B, itraconazole	*Aspergillus* spp., *Candida* spp., *Cryptococcus* spp.	Target the antioxidant system	Kim et al., 2012
Kojic acid	Amphotericin B	*Aspergillus fumigatus*, *Aspergillus terreus*, *Acremonium* sp., *Scedosporium* sp., *A. flavus*, *A. parasiticus*, *A. oryzae*, *A. niger*, *A. ochraceous*, *A. nidulans*, *P. expansum*, *P. chrysogenum*, *Penicillium glabrum*, *Penicillium chrysogenum*, *P. griseofulvum*, *P. italicum*, *S. cerevisiae*	Target the antioxidant system	Kim et al., 2013a
Octyl gallate (OG) 2,3-dihydroxybenzaldehyde (2,3-DHBA)	Pyraclostrobin	*Candida* spp., *Cryptococcus* spp.	Inhibiting mitochondrial respiratory chain	Kim et al., 2013b
Lopinavir	Azole drugs	*C. auris*, *C. albicans*, *C. tropicalis*, *C. krusei*, *C. parapsilosis*	Interfere with the glucose permeation and ATP synthesis	Eldesouky et al., 2020b
Allicin	Amphotericin B	*Candida albicans*	Vacuole-targeting fungicidal activity	Ogita et al., 2012
Pitavastatin	Fluconazole, voriconazole	*Candida albicans*	Targeting ABC efflux trasporter	Eldesouky et al., 2020
Thymol and other benzo analogs	Amphotericin B	*Aspergillus flavus*, *Aspergillus fumigatus*, *Aspergillus terreus*	Targeting cellular antioxidant system	Kim et al., 2008
Eight compounds with cyclobutene-dione (squarile) group	Fluconazole	*C. albicans*	Inhibit MFS efflux pump CaMdr1p	Keniya et al., 2015
*Alpinia officinarum* rhizome extract (AORE)	Cisplatin	–	Target the antioxidant system	Abass et al., 2018

Predominantly, natural phytocompounds can serve as a great therapeutic agent and precursor for the modification of structures to augment the antifungal activity of commercial antimycotic drugs. Several natural essential oils, plant extracts, and secondary metabolites have been applied as a chemosensitizer for the treatment of fungal infections along with the commercial antifungal drugs. Various plant extracts, grape seed extract, tea extracts, and essential oils have been used including carvacrol (Rao et al., 2010), anethole (Kubo and Himejima, 1991), cinnamaldehyde analogs, anisaldehyde analogs (Shreaz et al., 2013), benzol analogs (Zore et al., 2011), cinnamic acid (Faria et al., 2011), thymol, eugenol, methyeugenol (Braga et al., 2007; Ahmad et al., 2010), geraniol (Shin, 2003), oils from *Origanum vulgare*, *Agastache rugose*, *Euphorbia characias*, *Santolina chamaecyparissus*, *Pelargonium graveolens*, *Melaleuca alternifolia*, *Thymus vulgaris*, *Cinnamomum cassia*, and *Ocimum sanctum*. Apart from many terpenoids and phenols including farnesol, retigeric acid B, capisterone A and B, and schinol, propylgallate, octylgallate, epigallocatechin gallate greatly intensify the antifungal activity along with the commercial antimycotic drugs by targeting the resistance mechanisms such as interfering with ergosterol synthesis, β (1-3) glucan synthase enzyme, and efflux pump transporters (Shin and Kang, 2003; Giordani et al., 2004; Giordani et al., 2006; Rosato et al., 2009; Campbell et al., 2012). Application of safer chemosensitizing agent has potential efficacy against various human pathogenic mycoses.

## Antifungal Pipeline for Natural Chemosensitizers

Exploring the natural compounds possessing the antifungal activity has now been reinforced at global level. A plenty of natural compounds from plants could serve as an excellent chemosensitizers and needs to be studied in detail for their properties as well. *Aristolochia bracteolata* applied in traditional medicine have been proved to have potent antifungal activity against *Aspergillus niger*, *Aspergillus terreus*, *Penicillium notatum*, and *Rhizopus stolonifer* (Kavitha and Nirmaladevi, 2009). Isatin is an alkaloid compound, isolated from *Couroupita guianensis*, having a great fungicidal efficacy against *Asperillus niger*, *Aspergillus flavus*, *Aspergillus fumigatus*, *Candida albicans*, *Mucor oryzae*, and *Rhizopus indicus* that were recorded (Kavitha et al., 2013). *Trianthema portulacastrum* has also proven to be an herbal medicine to combat against various fungal pathogens, namely, *Aspergillus niger*, *Aspergillus fumigatus*, *Rhizopus*, and *Candida albicans* (Kavitha et al., 2014). Khanzada et al. (2021) have studied several edible medicinal plants such as *Cinnamomum zeylanicum*, *Cinnamomum tamala*, *Amomum subulatum*, *Trigonella foenumgraecum*, *Mentha piperita*, *Coriandrum sativum*, *Lactuca sativa*, and *Brassica oleraceae* var. italic to validate their antifungal efficacy against *Fusarium solani*, *Aspergillus niger*, *Aspergillus flavus*, and *Mucor* sp.; among them, *Cinnamomum zeylanicum* and *Cinnamomum tamala* have exhibited greatest antifungal activity that might be due to the presence of rutin and kaemferol. *Vitis vinifera* contains phenolics and flavonoids in their extracts in sufficient amount and thus, in turn, exhibits strong antifungal activity against numerous clinical pathogens (Simonetti et al., 2020). Nearly 23 hydroethanolic extract of medicinal plants were tested on *Candida albicans*, *Cryptococcus neoformans*, and *Cryptococcus gatti*. Among them, *Poincianella pyramidalis*, *A. occidentale*, *Anadenanthera colubrina* var. *cebil*, *Myracrodruon urundeuva*, and *Mimosa oftalmocentra* show potential fungicidal activity against all kinds of strains (Silva et al., 2020). *Aloe megalacantha* is a traditional medicinal plant employed for the treatment of various symptoms and infections including wound, dandruff, malaria, diabetes, impotence, colon cleansing, amoeba, ascariasis, abdominal pain, urine retention, snake bite, and evil eye. This plant extract has exhibited highest inhibitory profile against *C. albicans*, *C. glabrata*, *C. tropicalis*, and *C. krusei* (Asmerom et al., 2020). By considering the fate of the medicinal plants and their compounds, it can be exploited as great chemosensitizers to sensitize the microbes to commercial antifungal drugs.

## AFP From *Aspergillus giganteus*

Natural compounds from plant origin or microbial origin could serve as great platform for enhanced fungicidal activity. Further, deep exploration into antifungal protein might provide a possible breakdown of resistance mechanism counteracted by pathogenic fungi, ultimately augmenting the strong antimycotic activity. The antifungal protein AFP is a secretory protein from a filamentous fungi *Aspergillus giganteus*, which are small, basic, and cysteine-rich proteins that endeavor the future antimycotic strategies due to their specific inhibitory potential on several filamentous and non-filamentous fungal pathogens (Krishnamurthy et al., 2020; Ravindhiran et al., 2021). β-barrel topology in AFP is composed of five antiparallel β-strand, could be stabilized by four disulfide bridges, and contributes to the extreme resistance to heat and protease degradation. Nearly 12 lysine residues can be partially involved in the formation of cationic site such as K9, K10, and K32, due to the positive net charge of AFP. After that, the cationic site conjugates with the hydrophobic domains, namely, Y29, V30, Y45, and V50 furnish the amphipathic nature of AFP. Paradoxically, the AFP can easily traverse and perturb the plasma membrane for the suppression of growth of numerous filamentous fungal pathogens. Furthermore, wide range of functional properties make AFP as a prime candidate for the treatment of fungal infections: i) AFP is found to be resistant to heat and protease degradation; ii) it is synthesized from *Aspergillus giganteus* by a sustainable way; iii) microliters of AFP can be effective against several fungal pathogens (Moreno et al., 2005; Barakat et al., 2010). *afp* gene is encoded for the AFP from *Aspergillus giganteus*, and it had been widely applied to suppress resistance mechanisms in various fungal pathogens. In addition, AFP enters into the nucleic acids of host cell and causes charge neutralization and condensation of the DNA molecule and thus, in turn, produces strong antifungal activity (Narvaez et al., 2018).

**Figure 4:**
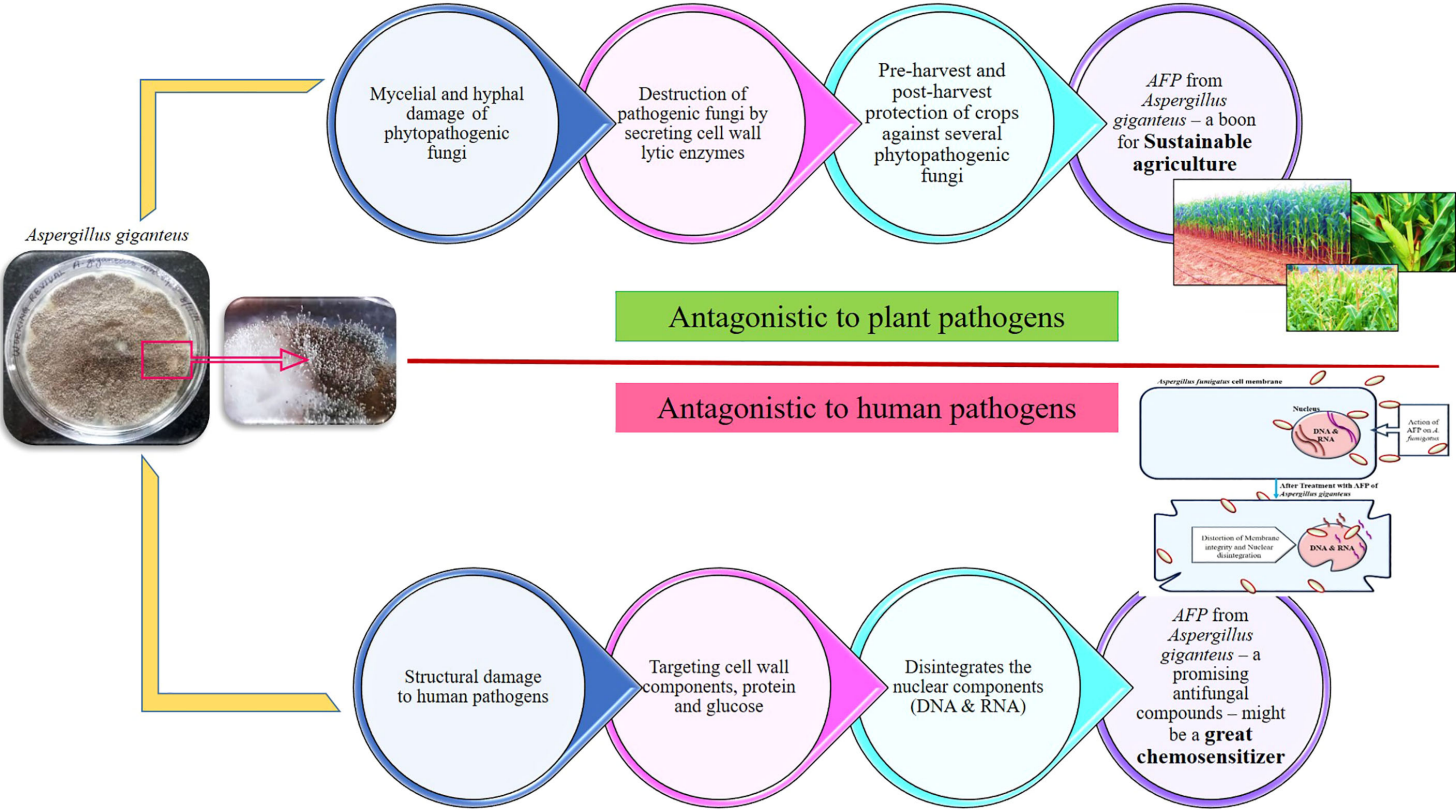
Antagonistic mechanism of *AFP* from *Aspergillus giganteus* on plant and human fungal pathogens. As a biocontrol agent, it selectively inhibits and kills the growth of plant fungal pathogens by secreting specific cell-wall degrading enzymes namely, amylase, protease, β-1,3 glucanase, chitinase, and cellulase. As antifungal compounds in clinical area, it could possibly perturb the membrane of human fungal pathogens by interfering with the cell wall components and nuclear components. AFP from *Aspergillus giganteus* might be a promising biocontrol agent for sustainable agriculture and optimistic novel compounds for the treatment of systemic fungal infections. In addition to their remarkable values, it could be a potent chemosensitizer to sensitize the plant and human fungal pathogens to commercial fungicides and antimycotic drugs.

Earlier studies in our laboratory have proved the efficacy of AFP protein from *Aspergillus giganteus* on various human pathogens, including *Aspergillus fumigatus*, *Aspergillus flavus*, *Cryptococcus neoformans*, and *Candida albicans* (Krishnamurthy et al., 2020; Ramya and Kavitha, 2021; Ravindhiran et al., 2021). Not only a human pathogen but also a phytopathogen has also been greatly governed by AFP. Aflatoxigenic *Aspergillus flavus* in corn at pre-harvest stage was completely eradicated by the application AFP in the field grown corn. The optimization for the carbon source, nitrogen source, pH, temperature, and water activity to enhance the AFP production and inhibition toward both phytopathogenic and zoopathogenic fungi were evaluated (Krishnamurthy et al., 2020). Virulence proteins of *Aspergillus fumigatus*, UDP-N-acetylglucosamine pyrophosphorylase (6TN3), N-myristoyl transferase (4CAW), and Chitinase (2A3B) are cell wall proteins, actively participated in the virulence mechanism in the host. In *Cryptococcus neoformans*, the target proteins are Farnesyl transferase (3SFX), Ionisine 5′-monophosphate dehydrogenase (4AF0), Ribokinase (6CW5), Serine/Threonine-protein phosphate 2B catalytic subunit A1 (6TZ8), Choline kinase (6WHP), *Candida albicans* Dihydrofolate reductase (1AI9), Myristoyl-CoA: protein N-Myristoyl transferases (1IYL), Secreted aspartyl proteases (1ZAP), and Lanosterol 14-α demethylase (5V5Z) are the potential virulence proteins responsible for the pathogenesis in the host. *In silico* prediction by molecular docking studies have proved that the antifungal proteins from *Aspergillus giganteus* and their derivatives has good interaction with the virulence proteins of *Aspergillus fumigatus*, *Candida albicans*, and *Cryptococcus neoformans* (Ravindhiran et al., 2021). Mechanism of action of *afp* on both plant and human fungal pathogens is depicted in **Figure 4**. AFP selectively inhibits the growth of fungal pathogens without affecting the host. AFP from *Aspergillus giganteus* has gained a much attention due to its application in medicinal panacea. AFP can act as a great chemosensitizer to make the pathogenic fungus susceptible to the commercially available antimycotic drugs. Co-application of chemosensitizing agents with AFP will alleviate the health and environmental associated risk by their unique underlying mechanisms. This could be a promising approach for the minimal amount of commercial drugs and can alleviate the burden of fungal infections at global level (Kim et al., 2008).

## Mode of Action of AFP From *Aspergillus giganteus*

Filamentous fungi are posing a petrifying threat in human; among them, *Aspergillus* spp. have been recorded with high mortality and morbidity rate, causing aspergillosis. Indistinguishably, *Aspergillus* and *Fusarium* spp. have been the rationality for the severe crop losses. However, the limited applications of the existing antimycotic drugs, a prerequisite for the efficient and economical antifungal agents, are highly preferable. Hence, the development of drugs with desired properties including sustainability, targeted actions, enhanced efficacy, and low toxicity is of major concern.

**Figure 5:**
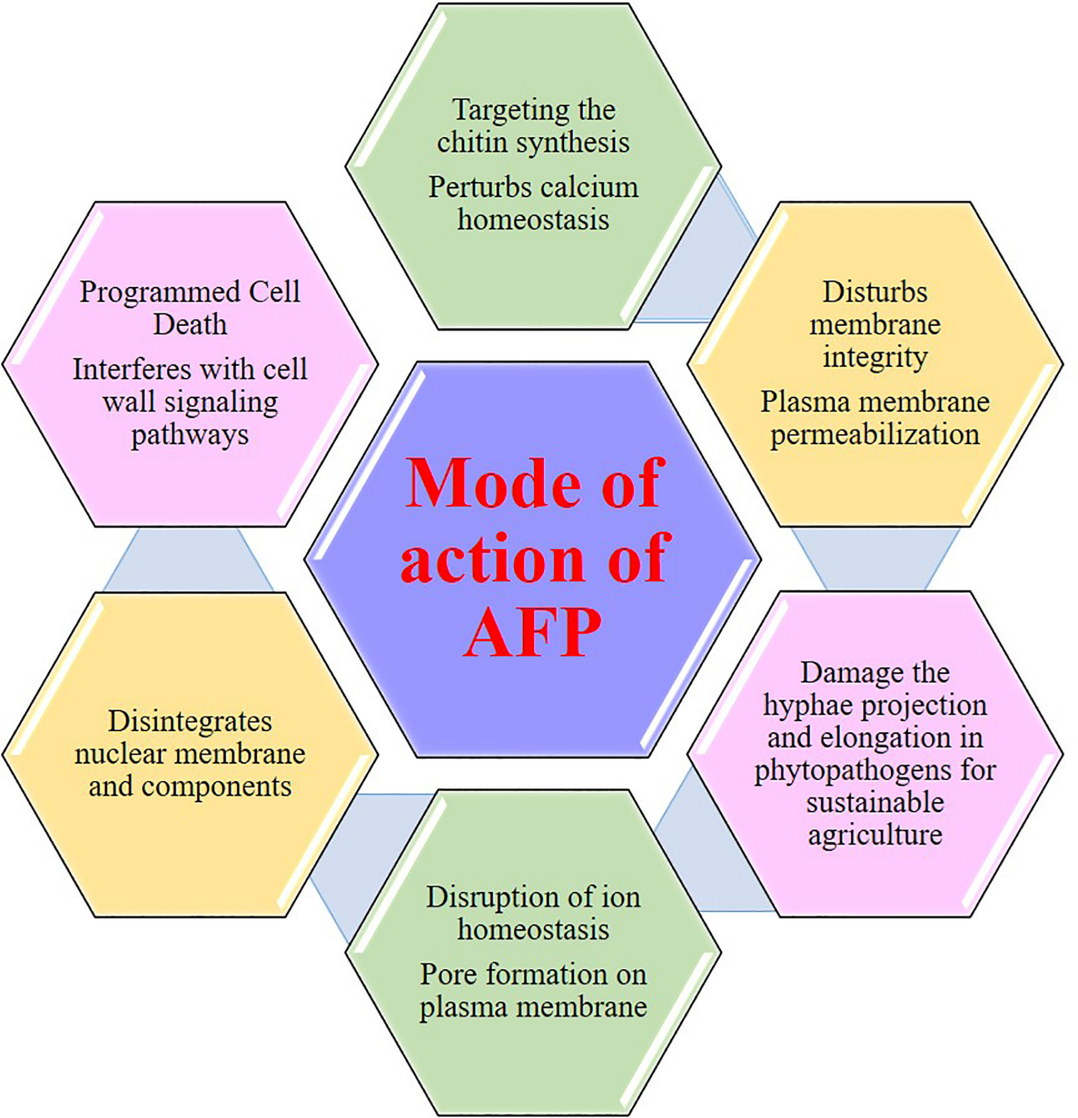
The versatile mode of action of AFP of *Aspergillus giganteus*.

Deciphering the mode of action of antifungal compounds is an important aspect to improve their efficacy to fight against the pathogens and interfere in the development of antifungal resistance mechanisms and amplification of their applications in therapeutics and in agriculture. Generally, AFP from *Aspergillus giganteus* can act on several cellular targets to exert their potential activities to eradicate the pathogens. In this regard, here, it throws light on the mode of action of AFP and harnesses their potential efficacy on controlling the pathogenic progressions. As noted above, the AFP has abundantly produced from *Aspergillus giganteus* with small (51 amino acids) and cationic structures. Minimal protein concentration ranging from 1 to 20 µM is enough to inhibit the growth of *Aspergillus* and *Fusarium* spp. Moreover, it does not exhibit cytotoxic and immunogenic effects on any types of mammalian cells, which attribute to their exemplary potential to act as a drug lead compounds. The mode of action of AFP from *Aspergillus giganteus* is explained in **Figure 5**.

AFP accumulates into the cell wall of pathogenic *Aspergillus niger* and is localized at the plasma membrane, causing severe membrane alterations in the pathogens as observed by electron microscopy. Nevertheless, this is the first AFP localized at the cell wall and plasma membrane identified so far. Thus, it could be studied extensively for the utilization of their remarkable targeting potential. Hagen et al. have studied the interaction of AFP with chitin biosynthesis pathway in pathogens. However, they found that N-terminal part from 1 to 33 amino acid is essential for efficient target binding and have a positive result on the production of truncated protein with the introduction of C14S, C26S, and C28S substitutions. From their studies, they have come to the conclusions that AFP can able to disturb the transport of chitosomes to the hyphae, control the release of chitin synthases from chitosomes, restrict the localization and anchoring of the enzyme to the plasma membrane for the chitin synthesis, and directly inhibit the chitin synthase enzyme activity or binding to the precursor molecules or to the polymerized chitin molecules. In their study, AFP has preferentially interfered with the chitosomes and class III and V chitin synthases enzymes. Finally, the membrane localization interaction that has occurred between chitin synthases and AFP contributes to the stretching of the plasma membrane, thus, significantly reducing the membrane integrity of the pathogens (Hagen et al., 2007).

Different culture conditions and protocols might have an influence on the antagonistic properties of the AFP from *Aspergillus giganteus*. For example, the MIC of AFP tested on agar dilution method is 20 times higher than that of broth microdilution methods. Sensitivity of the pathogens to AFP is determined due to the interactions with a membrane-based target, and it had been confirmed by the localization experiments. The studies confirmed that the AFP was constrained with in the hyphae and spheroplasts, which has been proved by FITC labeling and immunofluorescence staining, and both the methods have exhibited that the AFP preferentially binds with the plasma membrane causing permeabilization. SYTOX green assay has detected the membrane permeabilization caused by AFP in sensitive fungi, but it can be achieved only when high concentration of AFP is treated, i.e., higher than MIC (1 µg/ml; but for membrane permeabilization 10 µg/ml is needed). Conclusively, the permeabilization of plasma membrane is achieved due to the interactions of cation with the negatively charged target site of AFP and the inhibitory potential of AFP could be attained by receptor-mediated binding mechanisms (Theis et al., 2003).

The mode of action of AFP against rice blast fungus *M. grisea* susceptibility was studied in plant and animal cells. The electron microscopic results have proved the AFP-induced cellular damage and pore formation on the plasma membrane was observed in treated *M. grisea*. The mechanism of action involves disruption of plasma membrane and permeabilization of the pathogens. Congo-red staining reveals the significant deposition of chitin at the hyphal tip and leads to no growth of hyphal elongation. Along with that, it interferes with the nucleic acids, causing neutralization and condensation, leading to cell death pertaining to their potential antifungal activity that was confirmed by Alexa-labeled SYTOX green uptake assay (Moreno et al., 2006). The antifungal properties of AFP promote the neutralization and condensation of DNA molecules, which were exhibited by electrophoretic mobility and EtBr displacement assay. Because AFP is an extracellular protein, it exerts their antagonistic function on phytopathogenic fungi on rice by binding to the membrane and nuclear targets of pathogen and also providing protections to the plants (Pozo et al., 2002). The microscopic observations revealed that the AFP has effectively reduced the hyphal elongation and swollen hyphal tip and attributes to the potential antifungal activity of *Aspergillus giganteus* on *Botrytis cinerea*. Botrytis blight is the disease caused by *B. cinerea* on ornamental plants and is responsible for the major crop loss. An additive effect of AFP with Cecropin A was highly measured when incubated the plants with the compounds at different time intervals. This study has claimed that the AFP is a promising biocontrol agent for sustainable agricultural practices (Moreno et al., 2003). Not only in medicine and agriculture, the prime application of AFP is also recorded in food industries for juice extraction and clarification. The polygalacturonases are pectinolytic enzymes that are used for the hydrolysis of pectin-cell wall back bone for juice preparation. The enhanced polygalacturonases activity was observed in presence of medium containing β-mercaptoethanol, Co^2+^, Mn^2+^, ${\text{NH}}_{4}^{+}$ , Na^+^, and dithiotheritol; thus, it could be a good alternative for pectinase production in food industries (Pedrolli and Carmona, 2010).

AFP_NN5353_ is a protein secreted by *Aspergillus giganteus*, a defense-like protein, rich in cytosine residues. The protein is known to inhibit the growth and germination of plant and human fungal pathogens. This protein rapidly elevates the Ca^++^ resting level in the fungal cell, and prolonged perturbation of Ca^++^ homeostasis caused programmed cell death in fungal pathogens. The studies revealed the interference of AFP with the cell wall signaling pathways and inhibition of fungal pathogens (Binder et al., 2011).

## Conclusion and Future directions

Global burden of fungal infections is of significant threat, and a number of treatment strategy has been emphasized to treat fungal infections. The rate of development of new antifungal drugs is unsatisfactory, and it could not fulfill the future demands. This should be taken under consideration as several patients are prone to severe fungal infections. Many fungal species have evolved resistance toward many commercial antimycotic drugs by several aforementioned mechanisms. Exploring the various aspects involved in antifungal resistance mechanisms will be a great approach of curing global incidence of fungal infections.

By virtue of the facts and factors, exploring chemosensitization strategy could be an excellent alternative approach for making the drug susceptible for fungal pathogens. Antifungal protein from *Aspergillus giganteus* has been proved to have an intense level of inhibition against several human and agricultural mycoses.

Because AFP has shown greater antifungal activity against both filamentous and non-filamentous fungal pathogens, combination with commercially available antibiotics could perturb the resistance mechanism to augment the fungicidal activity of antimycotic drugs. The cytotoxic profile of AFP conveys that it is found to be safe, and even a lower amount of AFP can be effective in inhibiting several pathogens.

Therefore, we find that AFP from *Aspergillus giganteus* recorded with promising antifungal activity against several pathogens has been proven to be a new kind of chemosensitizer. Conclusively, the AFP has the chemosensitizing attribute to enhance the sensitivity of conventional antifungal drugs. Further, the chemosensitization strategy is required to study extensively using *in vitro* and *in vivo* model system to validate their potential augmenting fungicidal capacities of existing antifungal drugs.

## Author Contributions

KD conceptualized and planning the coherency of the manuscript, and all other authors contributed to the organization, review, editing, and preparation of the paper. RR and KS completed all figures and JNS provided critical data. All authors contributed to the article and approved the submitted version.

## Conflict of Interest

The authors declare that the research was conducted in the absence of any commercial or financial relationships that could be construed as a potential conflict of interest.

## Publisher’s Note

All claims expressed in this article are solely those of the authors and do not necessarily represent those of their affiliated organizations, or those of the publisher, the editors and the reviewers. Any product that may be evaluated in this article, or claim that may be made by its manufacturer, is not guaranteed or endorsed by the publisher.

## Abbreviations

AFP, antifungal protein; VOR, voriconazole; AmB, amphotericin B; POS, posaconazole; MICA, micafungin; ITR, itraconazole; ANI, anidulafungin; CAS, caspofungin; AmA, amphotericin A; 5-FC, 5-fluorocytosine; ABC, ATP-binding cassette transporters; MFS, major facilitator superfamily transporters; NBD, nucleotide-binding domains; TMD, transmembrane domains; TMS, transmembrane α-helix; MDR, multi-drug resistance; 5FU, 5-fluorouridine; ALS, agglutinin-like sequence; SAPs, serine aspartate proteases; LIP, lipases; PLB, phospholipases; DNA, deoxyribonucleic acid; MIC, minimum inhibitory concentration; FITC, fluorescein isothiocyanate.
